# Research progress of sea buckthorn (*Hippophae rhamnoides L.*) in prevention and treatment of cardiovascular disease

**DOI:** 10.3389/fcvm.2024.1477636

**Published:** 2024-10-18

**Authors:** Yumeng Chen, Weiwei He, Hanjing Cao, Zhenzhen Wang, Jiping Liu, Bin Wang, Chuan Wang

**Affiliations:** ^1^ Department of Pharmacology, Shaanxi University of Chinese Medicine, Xianyang, China; ^2^ Department of Physiology, Shaanxi University of Chinese Medicine, Xianyang, China; ^3^ Department of Nursing, Shaanxi University of Chinese Medicine, Xianyang, China

**Keywords:** sea buckthorn, phytochemistry, pharmacology, clinical application, cardiovascular disease

## Abstract

Sea buckthorn (*Hippophae rhamnoides L.*) contains a variety of biologically active compounds, including flavonoids, terpenoids, polysaccharides, organic acids, volatile oils, and vitamins. It has been demonstrated to be effective in the treatment of cardiovascular disorders. In this paper, we evaluated the pharmacological effects of sea buckthorn in cardiovascular diseases through preclinical studies, and revealed the mechanism of action of the active components in sea buckthorn in cardiovascular diseases, including anti-inflammatory, lipid oxidation regulation, antioxidant, vascular function modulation, anti-platelet aggregation, autophagy, intestinal microorganism regulation, and cell apoptosis reduction. In clinical trials, sea buckthorn was proven to be effective in managing lipid metabolism, blood pressure, and blood glucose levels in patients. We also extensively reviewed the safety of sea buckthorn medicine and its toxicity to numerous organs. To summarize, sea buckthorn has a beneficial effect on cardiovascular disease and may give a novel strategy for clinical intervention and therapy. This paper summarizes the phytochemistry, pharmacology, clinical applications, safety, and toxicity of sea buckthorn in order to better understand the mechanism of action of the various bioactive components in sea buckthorn, investigate its medicinal potential, and provide more options for the treatment of cardiovascular diseases.

## 1 Introduction

Cardiovascular disease (CVD) has long been an important cause of harm to humans. Owing to the rapid aging of the global population and the adoption of unhealthy lifestyles by residents, the incidence of CVD increasing. Cardiovascular diseases affect more than 523 million people worldwide (1). The development of CVD is multifaceted and multifactorial, and is influenced by factors such as the environment, poor lifestyle habits, diet, mental health, and genetics. Many nontraditional risk factors as well as biomarkers may be linked to the development of CVD in addition to traditional risk factors. Examples of such biomarkers include troponin, natriuretic peptide, oxidative stress biomarkers, and hemoglobin (2). A body of evidence indicates that patients with CVD are at an elevated risk of experiencing a decline in kidney function and the development of kidney disease. Moreover, there is a significant incidence of CVD among individuals with chronic kidney disease (3, 4). Currently, statins, aspirin, β-blockers, calcium channel blockers, ARBs, ACEIs, and nitrates are commonly used in the clinic to prevent and treat CVD. However, with their widespread use in the clinic, adverse effects have gradually emerged. Among statins, simvastatin and atorvastatin are associated with a greater risk of rhabdomyolysis (5). Patients treated with ACEIs have a relatively high incidence of bronchospasm and cough (6). In addition, beta-blockers inhibit sympathetic excitation and mask hypoglycemic symptoms and are contraindicated in patients with acute left heart failure, bronchial asthma, asymptomatic hypoglycemia, and atrioventricular block of degree II or greater (7). Therefore, finding safer and more effective drugs is a key issue that needs to be addressed at present. Chinese medicines help to comprehensively regulate the complex pathological process of CVD through multicomponent and multitarget mechanisms of action, which may lead to new ideas and methods for CVD prevention and treatment.

Sea buckthorn, a deciduous shrub primarily found in Asia, Europe, and North America, belongs to the *Hippophae* family. Its application originated in ancient Greece, where the ancient Greeks found that horses’ coats shone brighter after eating sea buckthorn berries, so they named it “*Hippophae rhamnoides L.*”, which means “the tree that makes horses shine”. From a broader perspective, the origin of sea buckthorn is thought to be between the Eastern Himalaya and the Hengduan Mountains (8). Sea buckthorn grows in arid and cold windy areas and has drought-resistant properties, which gives it an advantage in managing geological disasters such as desertification and arsenic sandstone (9).

The plant sea buckthorn is used for many purposes, such as food production and medicine. It stands out for having a high nutritional and medicinal value. Sea buckthorn contains a diverse array of nutrients, such as vitamins, proteins, amino acids, organic acids, and inorganic elements (10). The medical history of sea buckthorn in China dates back to the Tang Dynasty, when it was first mentioned in the Tibetan medical literature. It was later documented in Mongolian and Uyghur medicine (11). As early as 900 B.C., it was discovered that sea buckthorn could be used to treat stomach ulcers, asthma, skin burns and CVD (12). Recently, academic professionals have been delving into the exploration of sea buckthorn, revealing promising therapeutic benefits in the treatment of multiple diseases through a range of preclinical and clinical studies. In addition, its effect on CVD is the focus of this paper. This paper summarizes the phytochemistry, pharmacology, clinical application and safety of sea buckthorn in CVD. It is hoped that this study can provide more references for the subsequent aspects of sea buckthorn in the treatment of CVD.

## 2 Phytochemistry

Various bioactive components of sea buckthorn berries, leaves and roots have potential medicinal and economic value. Sea buckthorn notably contributes to the therapeutic management of CVD, effectively alleviating symptoms associated with conditions such as atherosclerosis (AS) and hypertension. To clarify the chemical components contained in sea buckthorn, researchers have extracted and isolated the active components from various parts of sea buckthorn for research. Over one hundred compounds, such as flavonoids, terpenoids, steroids, phenols, essential oils, vitamins, amino acids, and trace minerals, have been discovered and recognized in different components of sea buckthorn. By conducting numerous animal studies, researchers have shed more light on the active components in sea buckthorn that have a significant impact.

Flavonoids constitute the primary bioactive constituents of sea buckthorn. More than 90 flavonoids, including flavonols, flavanols, dihydroflavonoids, anthocyanins and chalcones, have been extracted and isolated from sea buckthorn (13). Flavonols are the main components of flavonoids, which are mainly derivatives of isorhamnetin and quercetin. Among these compounds, isorhamnetin, quercetin, and kaempferol exist in glycosylated forms, which combine with glucose, rhamnose, arabinose, and rutinose to form glycosides. Isorhamnetin-3-O-glucoside-7-O-rhamnoside, isorhamnetin-3-O-rutinoside and kaempferol-3-O-hexoside-7-O-rhamnoside are the three main flavonol glycosides (14). The flavonoid composition and content of different parts of sea buckthorn vary slightly. Flavonols (isorhamnetin, populin, kaempferol, sanguinarine, quercetin) are mainly concentrated in sea buckthorn berries; flavanols (catechin, epicatechin, epigallocatechin, gallocatechin) are found only in sea buckthorn leaves; and dihydroflavonoids (pinostrobin, naringenin, nerolidin, dihydromyricetin, sanguinarin, dihydroquercetin), proanthocyanidins (proanthocyanidin B1, proanthocyanidin B2), and chalcones are found in the stems of sea buckthorn. and dihydroquercetin), proanthocyanidins (proanthocyanidin B1, proanthocyanidin B2) and chalcones (15). Sea buckthorn leaves boast a higher flavonoid content than sea buckthorn berries, whereas the varieties of flavonoids found in berries surpass those in the leaves (16). The total flavonoid content of each part of sea buckthorn, in descending order, was as follows: leaf, pulp, whole fruit pomace, pericarp and seed (17). The terpenoids in sea buckthorn are mainly triterpenoids, including oleanolic acid, ursolic acid and 2α-hydroxyursolic acid. Among them, high levels of ursolic acid are detected in sea buckthorn leaves, accounting for 46% of the average total triterpenoids (18). The steroids in sea buckthorn are predominantly sterol, ergosterol and lanosterol types (19). Baoru et al. analyzed sterols in the seeds, pulp/pericarp and berries of two subspecies of sea buckthorn from Finland and China (*sinensis* and *rhamnoides*) and reported that the seeds contained the highest amount of total sterols at 1,200–1,800 mg/kg, the berries were in the middle of the range, and the pulp/pericarp had the lowest amount. Sterols are predominantly glutenol, accounting for 57%–76% and 61%–83% of the sterols in seeds and pulp/pericarp, respectively (20). Sea buckthorn contains abundant phenolic compounds. It possesses potent antioxidant activity, and there is a substantial correlation between the antioxidant capacity and the total phenolic compounds. With the growth and development of sea buckthorn leaves, their total phenolic compounds and antioxidant capacity tend to increase (21). The volatile oil components are mainly found in the berries and seeds of sea buckthorn, with lower amounts in sea buckthorn leaves, and are mainly fatty acids, esters, alcohols and aliphatic compounds. The results of the current research suggest that palmitoleic acid is the dominant fatty acid in the pericarp of sea buckthorn berries, with palmitic acid following closely behind. In contrast, oleic acid was identified as the predominant fatty acid in sea buckthorn seeds, followed by palmitic and linoleic acids (22). Sea buckthorn is rich in vitamins A, B1, B2, C, D, E, carotene, folic acid, etc., in which the content of vitamin C is the highest, and it is known as “vitamin treasury”. The vitamin C content of sea buckthorn is influenced by the genetic background of sea buckthorn and the date of harvest, and the vitamin C content of the juice of the Chinese subspecies is 5–10 times greater than that of subsp. *rhamnoides* from Europe and subsp. *mongolica* from Russia (23). In addition, sea buckthorn contains many amino acids as well as trace inorganic elements, and berries and leaves contain similar species (16). We collated the sea buckthorn extracts and their active ingredients that have shown beneficial effects on CVD in previous studies (Table 1).

**Table 1:** Effect of sea buckthorn extract and its active ingredients on CVD.

Sample	Main active ingredients	Molecular formula	Content	Biological activity in CVD	Reference
Sea buckthorn powder cubes	Linolenic acid	C_18_H_30_O_2_	36.6 wt%	Mean arterial pressure↓, heart rate↓, TC↓, TG↓, glycosylated hemoglobin↓	(24)
	Linolic acid	C_18_H_32_O_2_	28.6 wt%		
	Oleic acid	C_18_H_34_O_2_	18.0 wt%		
	β-sitosterol	C_29_H_50_O	220 mg/100g		
	Rutin	C_27_H_30_O_16_	35 mg/100 g		
Sea buckthorn freeze-dried powder	Total polyphenols	–	6.55 ± 0.29 mg/g	Regulates intestinal microbiota and inhibits fat accumulation	(25)
	Total acid	–	14.38 ± 0.62 mg/g		
	Not mentioned	–	–	Activation of the AMPK/SIRT1 pathway	(26)
Aqueous extract powder of *H. rhamnoides* fruits	1,5-Dimethyl Citrate	C_8_H_12_O_7_	0.022 mg/g	IKKα/β↓, IκBα↓, NF-κBp65↓, iNOS↓, COX-2↓, IL-6↓, TNF-α↓	(27)
Sea buckthorn fruit oil extract	Not mentioned	–	–	p-AMPK and p-Akt proteins↑	(28)
Sea buckthorn fruit oil	Not mentioned	–	–	Free fatty acids↑, SREBP-1↓, FAS↓, ACC synthesis↓	(29)
Sea buckthorn pulp oil	SFA	–	33.909 ± 0.216%	Serum and liver TC↓, LDL-C↓; Increases the richness of the gut microbiota	(30)
	MUFA	–	58.918 ± 0.238%		
	PUFA	–	7.122 ± 0.028%		
	Total tocopherols	–	1,834.67 ± 91.82 mg/kg		
	Total phytosterols	–	11,233.66 ± 62.99 mg/kg		
Sea buckthorn seed oil	SFA	–	11.541 ± 0.111%		
	MUFA	–	24.280 ± 0.057%		
	PUFA	–	63.387 ± 0.032%		
	Total tocopherols	–	2,806.33 ± 14.22 mg/kg		
	Total phytosterols	–	9,809.67 ± 31.68 mg/kg		
	Fatty acids	–	99.7 wt%	TC↓, TG↓, LDL-C↓, HDL-C↑	(31)
	Carotenoids	–	350 mg/kg		
	Tocopherols/tocotrienols	–	1,389.9 mg/kg		
	Sterols	–	16,888 mg/kg		
	Total SFAs	–	12.07 ± 0.01%	Cholesterol absorption↓, SCFAs↑, alters the gut microbiota, HMG-CoA-R, ACAT2, MTP and ABCG8 genes↓, intestinal SCFAs↑	(32, 33)
	Total MUFAs	–	24.37 ± 0.03%		
	Total PUFAs	–	63.56 ± 0.03%		
	Phytosterols	–	1,530.91 ± 5.53 mg/100 g oil		
Sea buckthorn leaf extract	Isorhamnetin	C_16_H_12_O_7_	112.65 ± 5.75 μg/g	Inhibits cytotoxicity and ROS production	(34)
	Quercetin-3-galactoside	C_21_H_20_O_12_	423.39 ± 8.75 μg/g		
	Kaempferol	C_15_H_10_O_6_	46.43 ± 2.28 μg/g		
	Ellagitannins	–	259.6 ± 3.1 mg/g	Anti-adhesion	(35)
	Total flavonoid	–	74.7 ± 0.7 mg/g		
	Catechin and proanthocyanidins	–	7.2 ± 0.2 mg/g		
Butanol extract from sea buckthorn twigs	B–type proanthocyanidins and catechin	–	597.1 ± 10.2 mg/g	Inhibits platelet adhesion to type I collagen	
	Ellagic acid and its glycosides	–	22.4 ± 0.1 mg/g		
	Flavonoids	–	1.7 ± 0.4 mg/g		
Sea buckthorn flavonoids	Isorhamnetin	C_16_H_12_O_7_	392.22 mg/g	NO/PGE2↓, INOS/COX-2 mRNA↓; TNF-α↓, IL-6↓, IL-1β↓; Inhibit MAPK and NF-κB signaling pathways	(36)
	Quercetin	C_15_H_10_O_7_	124.03 mg/g		
	Kaempferol	C_15_H_10_O_6_	56.53 mg/g		
	Not mentioned	–	–	MDA↓, IL-1β↓, IL-6↓, TNF-α↓; SOD↑, GSH-Px↑	(37)
	Not mentioned	–	0.729 mg/g	Inhibit TLR4/IL-6/STAT3 pathway	(38)
	Not mentioned	–	–	Inhibits NLRP3 pathway	(39)
	Isorhamnetin	C_16_H_12_O_7_	284.95 mg/g	Promotes the conversion of cholesterol to bile acids and cholesterol efflux; Inhibits *de novo* cholesterol synthesis and accelerates FA oxidation	(40)
	Quercetin	C_15_H_10_O_7_	104.58 mg/g		
	Kaempferol	C_15_H_10_O_6_	24.15 mg/g		
	Not mentioned	–	–	Decreases NADPH oxidase, increases SIRT1 expression	(41)
	Isorhamnetin 3-O-glucoside-7-O-rhamnoside	C_28_H_32_O_16_	–	Antioxidant; Modulates the PI3 K/AKT-eNOS pathway	(42)
	Rutin	C_27_H_30_O_16_	–		
	Laricitrin 3-O-rutinoside	C_28_H_32_O_17_	–		
	Isorhamnetin	C_16_H_12_O_7_	25.4%	Regulates the expression of eNOS and LOX-1	(43)
	Quercetin	C_15_H_10_O_7_	57.7%		
	Not mentioned	–	–	Blocks VDC and ROC to reduce [Ca^2+^] levels	(44)
	Not mentioned	–	–	Up-regulated levels of beclin-1 and LC3 proteins	(45)
Phenylpropyl compounds from sea buckthorn	p-coumaric acid	C_9_H_8_O_3_	–	Downregulation of cleaved caspase-3 expression; Activates mitochondrial biogenesis and maintains mitochondrial function	(46)
	Chlorogenic acid	C_16_H_18_O_9_	–		
	Caffeic acid	C_9_H_8_O_4_	–		
	Ferulic acid	C_10_H_10_O_4_	–		
Sea buckthorn flavonoid powder	Not mentioned	–	–	Increases HL, LPL, lipase, SOD and GSH activity; MDA ↓	(47)
Total flavonoids from seed residues of *Hippophae rhamnoides L*.	Not mentioned	–	699.2 ± 18.4 g/kg	Serum TC↓, LDL-C↓; Liver TC↓, TG↓	(48)
Phenolic extract of sea buckthorn fruit	Flavonoids	–	214.04 mg/g	Interfere with arachidonic acid metabolism and ROS↓; inhibits thrombin proteolytic properties	(49, 50)
	Isorhamnetin 3-O-hexoside-deoxyhexoside	–	44.00 ± 0.35 mg/g		
	Isorhamnetin 3-O-hexoside	–	44.16 ± 0.08 mg/g		
Sea buckthorn polyphenol extract	Gallic acid	C_7_H_6_O_5_	–	LDH↓, CK-MB↓, Inhibits beclin-1 and LC3 proteins	(51)
	Proto catechuic acid	C_7_H_6_O_4_	–		
	(+)-catechin	C_15_H_14_O_6_	–		
	Caffeic acid	C_9_H_8_O_4_	–		
	Chlorogenic acid	C_16_H_18_O_9_	–		
	Ferulic acid	C_10_H_10_O_4_	–		
	p-coumaric acid	C_9_H_8_O_3_	–		
	Salicylic acid	C_7_H_6_O_3_	–		
	Cinnamic acid	C_9_H_8_O_2_	–		
	Quercetin	C_15_H_10_O_7_	–		
	Phlorizin	C_21_H_24_O_10_	–		
	Rutin	C_27_H_30_O_16_	–		
	Kaempferol	C_15_H_10_O_6_	–		
	Isorhamnetin	C_16_H_12_O_7_	–		
Sea buckthorn polyphenols	Not mentioned	–	89.98 ± 0.032%	Decreased mRNA and protein expression of eNOS and LOX-1; Alleviate ICAM expression	(52, 53)
Sea buckthorn Procyanidins	(−)-epicatechin gallate	C_22_H_18_O_10_	–	Inhibition of p38MAPK/NF-κB signaling pathway; Increases mitochondrial membrane potential and NO levels	(54)
	procyanidin B	C_30_H_26_O_12_	–		
	(+)-gallocatechin-(+)-catechin	C_30_H_26_O_13_	–		
	(+)-gallocatechin dimer	C_30_H_26_O_14_	–		
Sea buckthorn polysaccharide	Not mentioned	–	–	Increase the number of beneficial bacteria	(55)
	GalA	C_6_H_10_O_7_	–		
	Isorhamnetin	C_16_H_12_O_7_	–	IL-6↓, TNF-α↓, MPC-1↓; Regulation of TLR-4/IκBα/NF-κBp65; Inhibition of NF-κB and AP-1 expression; Activates the PI3K/AKT pathway	(56–59)
	Quercetin	C_15_H_10_O_7_	–	Blocks VDC and ROC to reduce [Ca2+] levels	(44)
	Isorhamnetin 3-O-beta-glucoside-7-O-alfa-rhamnoside	C_28_H_32_O_16_	–	Antioxidant	(60, 61)
	Isorhamnetin 3-O-beta-glucoside-7-O-alfa-(3*”'*-isovaleryl)-rhamnoside	C_33_H_40_O_17_	–	Anticoagulant, antiplatelet activity	

## 3 Pharmacology

### 3.1 Anti-inflammation

The inflammatory response is closely linked to the pathogenesis of CVD. Sea buckthorn is able to inhibit the formation and progression of CVD by suppressing the inflammatory response. The interaction of inflammatory cytokines in the inflammatory response determines the trend and final outcome of inflammation (59). Sea buckthorn flavonoid components inhibited LPS-induced NO/PGE_2_ production and iNOS/COX-2 mRNA expression. It reduces the production of TNF-α, IL-6 and IL-1β at the protein and mRNA levels (35). In another study, 1,5-dimethyl citrate extracted from sea buckthorn was shown to have anti-inflammatory effects on LPS-induced RAW264.7 mouse macrophages (28). In a rat model of exercise-related myocardial injury, the levels of MDA, IL-1β, IL-6, and TNF-α in rat myocardial tissues were significantly reduced, and the levels of SOD and GSH-Px were significantly increased by sea buckthorn flavonoid treatment (36). Isorhamnetin is an active component of sea buckthorn, and it has been reported that isorhamnetin inhibits the release of the inflammatory factors IL-6, TNF-α, and MCP-1 from THP-1 cells stimulated with LPS (53).

The inhibitory effect of sea buckthorn on inflammatory factors is closely associated with the expression of inflammation-related signaling pathways. TLR4 is closely related to the body's inflammatory response, and STAT3 can accelerate the development of inflammation by promoting the release of IL-6 via TLR4 (60). Sea buckthorn flavonoids reduce inflammation and alleviate atherosclerotic symptoms in atherosclerotic mice through the TLR4/IL-6/STAT3 pathway (37). In addition, sea buckthorn flavonoids blocked the activation of the MAPK (SAPK/JNK and p38) and NF-κB signaling pathways (35). The researchers isolated peripheral blood mononuclear cells from human blood and induced their differentiation into macrophages, followed by stimulation of macrophages with ox-LDL to form foam cells. Isorhamnetin administration reduced macrophage foam cell formation as well as CD36 and TLR-4 protein expression. *In vivo*, isorhamnetin treatment improved the expression of TLR-4 mRNA and the TLR-4 pathway in the aortic tissues of ApoE^−/−^ mice. Overall, isorhamnetin inhibits inflammation in ApoE^−/−^ mice by modulating the TLR-4/IκBα/NF-κBp65 pathway (54).

Adhesion molecules are widely used as proinflammatory markers involved in the adhesion and migration of inflammatory cells to damaged tissues and play important roles in the treatment of CVD (61). Isorhamnetin inhibited TNFα-induced apoptosis and upregulated the adhesion molecules ICAM-1, VCAM-1, and E-selectin by inhibiting the expression of NF-κB and AP-1. These findings suggest potential antiapoptotic and anti-inflammatory effects of isorhamnetin on TNFα-induced HUVECs (55).

**Figure 1:**
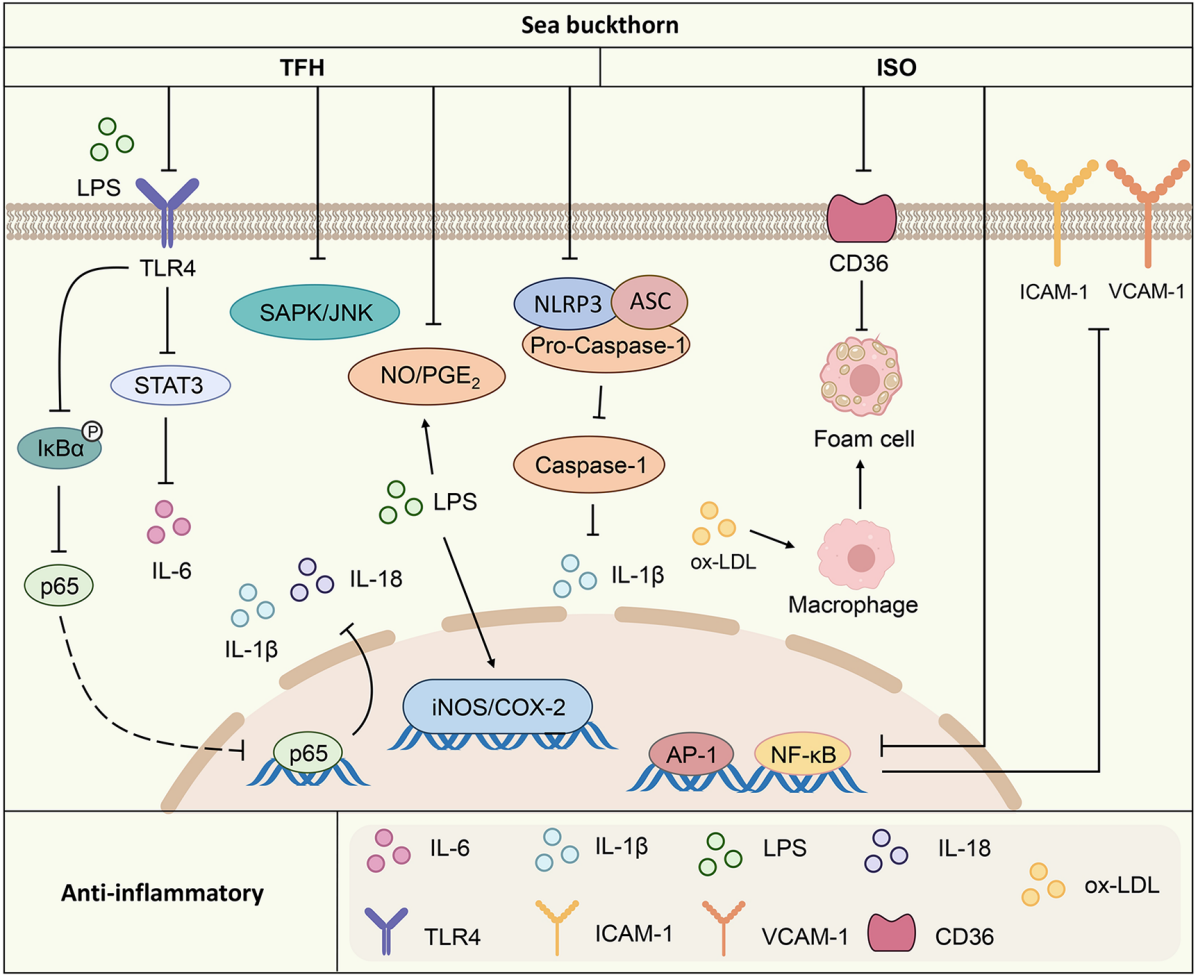
Anti-inflammatory mechanism of action of the active components of sea buckthorn. TFH, sea buckthorn flavonoids; ISO, isorhamnetin; LPS, lipopolysaccharides; TLR4, toll-like receptor 4; STAT3, signal transducers and activators of transcription 3; IκBα, inhibitor kappa B alpha; IL-1β, interleukin-1 beta; IL-18, interleukin-18; IL-6, interleukin-6; CD36, cluster of differentiation 36; ox-LDL, oxidized low-density lipoprotein; SAPK/JNK, stress-activated protein kinase/c-Jun N-terminal kinase; NO/PGE2, nitric oxide/Prostaglandin E2; AP-1, activating protein-1; NF-κB, nuclear factor kappa B; iNOS/COX-2, inducible nitric oxide synthase/cyclooxygenase-2; NLRP3, NOD-like receptor thermal protein domain associated protein 3; ASC, apoptosis-associated speck-like protein containing a CARD; Pro-Caspase-1, pro-cysteinyl aspartate specific proteinase-1; ICAM-1, intercellular cell adhesion molecule-1; VCAM-1, vascular cell adhesion molecule-1.

Overall, sea buckthorn inhibits the release of various proinflammatory factors. The inhibitory effects on inflammatory factors are associated mainly with pathways such as the TLR4/IL-6/STAT3, MAPK and NF-κB pathways. It also inhibits the inflammatory response by regulating the expression of the adhesion molecules ICAM-1 and VCAM-1 (Figure 1).

### 3.2 Regulation of lipid metabolism

A disturbance in lipid metabolism plays a fundamental role in the pathogenesis of AS. Sea buckthorn seed oil is a valuable source of unsaturated fatty acids, flavonoids, phytosterols and other beneficial compounds. Flavonoid extracts from sea buckthorn seeds can effectively reduce serum TC and LDL-C levels in mice fed a high-fat diet, while liver TC and TG levels are also increased (45). The administration of sea buckthorn seed oil to a rabbit model fed a high cholesterol diet resulted in a reduction in plasma cholesterol and LDL-C levels, accompanied by an increase in HDL-C levels. This treatment also led to notable decreases in the atherosclerotic index and LDL/HDL ratio. These findings suggest that sea buckthorn seed oil has a significant antiatherosclerotic effect (32). Moreover sea buckthorn pulp and seed oil can regulate the intestinal microbiota and ameliorate lipid metabolism disorders (31). One study reported that intervention with sea buckthorn freeze-dried powder alleviated circulating lipid levels and improved insulin sensitivity in mice on a high-fat diet. In addition, sea buckthorn freeze-dried powder activated the AMPK/SIRT1 pathway and improved beige adipocyte formation in white adipose tissue, which in turn intervened in lipid metabolism (27). Sea buckthorn flavonoid extract upregulated the mRNA expression of PPARγ, PPARα, ABCA1 and CPT1A and downregulated SREBP-2. In addition, sea buckthorn flavonoids may promote the conversion of cholesterol to bile acids and cholesterol efflux, inhibit cholesterol synthesis and accelerate the oxidation of fatty acids, which in turn may ameliorate hyperlipidemia (38).

**Figure 2:**
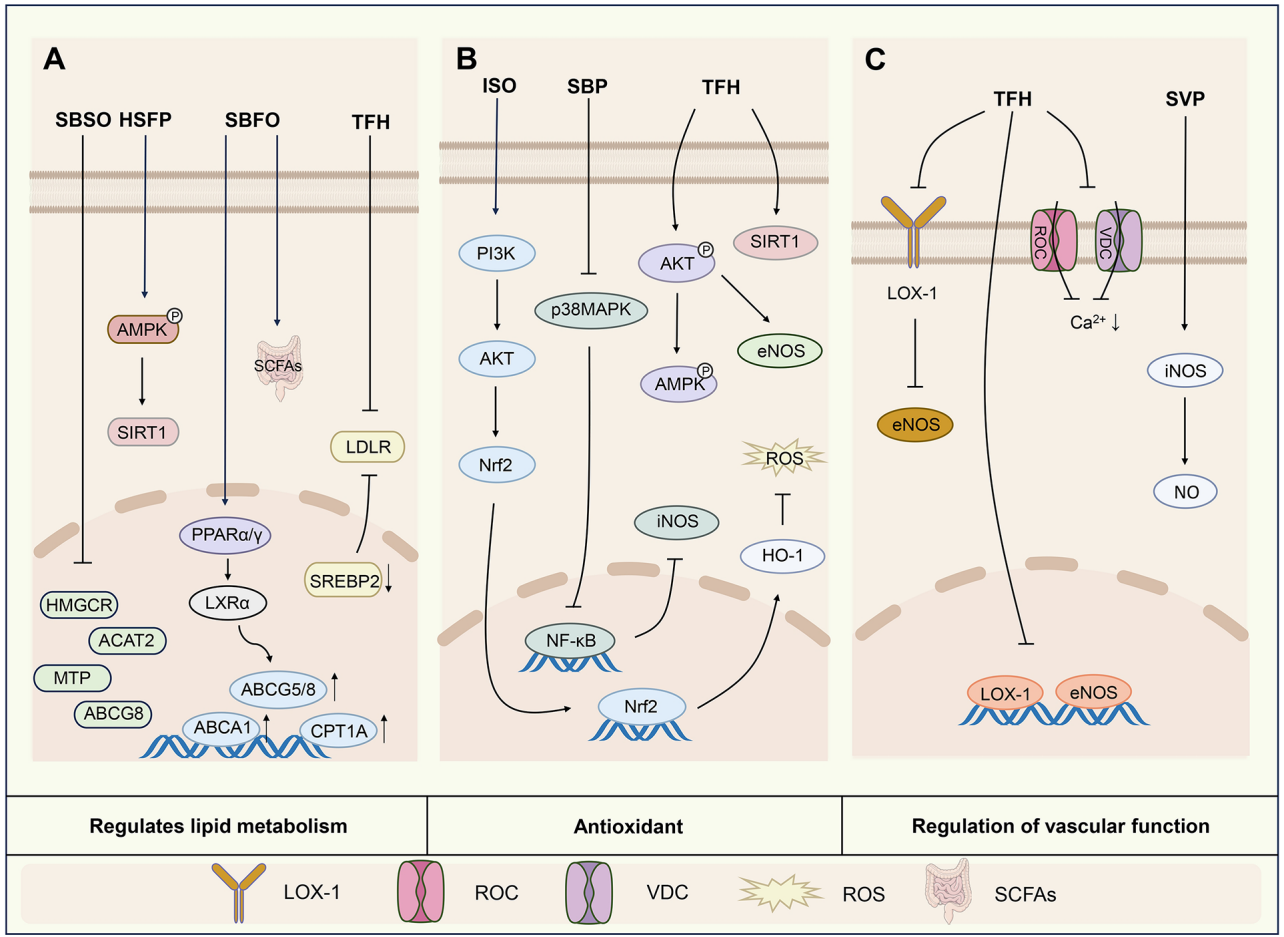
Mechanisms of action of active components of sea buckthorn in regulating lipid metabolism **(A)**, antioxidant **(B)** and regulating vascular function **(C)**. SBSO, sea buckthorn seed oil; HSFP, sea buckthorn freeze-dried powder; SBFO, sea buckthorn fruit oil; SBP, sea buckthorn procyanidins; SVP, sea buckthorn berries; AMPK/SIRT1, adenosine 5′-monophosphate-activated protein kinase/silent information regulator 1; ACAT2, acetyl-CoA acetyltransferase 2; MTP, microsomal triacyglycerol transport protein; ABCG8, ATP-binding cassette transporter subfamily G member 8; PPARα/γ, peroxisome proliferator-activated receptor α/γ; LXRα, liver X receptor α; CPT1A, carnitine palmitoyltransferase1; SREBP-2, sterol-regulatory element binding protein 2; LDLR, low-density lipoprotein receptor; SCFAs, short-chain fatty acids; PI3K/AKT, phosphatidylinositol 3-kinase/protein kinase B; Nrf2, nuclear factor-erythroid 2 related factor 2; HO-1, heme oxygenase 1; ROS, reactive oxygen species; p38MAPK, p38 mitogen-activated protein kinase; eNOS, endothelial nitric oxide synthase; LOX-1, lectin-like oxidized low-density lipoprotein receptor 1; ROC, receptor-operated calcium channels; VDC, voltage-dependent calcium channels.

Therefore, previous studies have demonstrated that sea buckthorn can regulate blood lipid levels and modulate the gut microbiota to improve lipid metabolism. It has also been associated with activation of the AMPK/SIRT1 pathway and upregulation of the expression of LXRα, ABCA1, ABCG1 and PPARγ (Figure 2A).

### 3.3 Antioxidants

The development of AS is significantly influenced by oxidative stress, which is caused by an imbalance between the production and degradation of ROS. This process plays a pivotal role in accelerating the progression of AS by promoting the oxidative modification of LDL to form ox-LDL (62, 63). Sea buckthorn flavonoids are able to increase the activities of hepatic lipase (HL), lipoprotein lipase (LPL), lipase, SOD, and GSH and reduce MDA levels. It has shown hypolipidemic and antioxidant effects (44). Sea buckthorn fruit oil extract promotes fatty acid oxidation by phosphorylating the AMPK and AKT proteins. In addition, non-HDL-C levels are significantly increased, and oxidative stress is alleviated (29). HO-1 is a target protein downstream of Nrf2. When an organism is stimulated, HO-1 expression is upregulated through the regulation of Nrf2, thus exerting antioxidant effects. Activation of the PI3K/AKT signaling pathway has been demonstrated to result in further activation of Nrf2 (64). Isorhamnetin has been reported to activate the PI3K/AKT pathway and increase HO-1 expression, resulting in a significant improvement in atherosclerotic plaque size (56).

Kumar et al. induced Raw264.7 mouse macrophages with tert-butyl hydroperoxide to increase cytotoxicity and ROS production and decrease the mitochondrial membrane potential. After treatment with sea buckthorn leaf extract, sea buckthorn extract inhibited cytotoxicity and ROS production and maintained antioxidant levels similar to those in control cells (33). Endothelial cell dysfunction is one of the major contributors to CVD, and amelioration of endothelial damage due to oxidative stress can reduce the likelihood of CVD (65). Flavonoids from sea buckthorn significantly inhibited oxidative stress-induced cellular damage, maintained endothelial cell integrity and function, and acted through the PI3K/AKT-eNOS pathway (39). Studies have demonstrated the positive impact of proanthocyanidins as powerful antioxidants in ameliorating endothelial damage (66, 67). Sea buckthorn proanthocyanidins increased the mitochondrial membrane potential and NO level in palmitic acid-induced oxidative damage in HUVECs and reduced LDH leakage to ameliorate oxidative damage. Moreover, sea buckthorn proanthocyanidins were shown to have a protective effect on HUVECs by inhibiting the p38MAPK/NF-κB signaling pathway. Additionally, the effects on LOX-1, eNOS and iNOS protein expression were observed (51).

The above studies have shown that sea buckthorn strongly regulates oxidative stress. This ability to regulate oxidative stress may be related to the regulation of oxidative stress indicators, activation of the PI3K/AKT pathway, regulation of the levels of phosphorylated AMPK and AKT proteins, reduction of NADPH oxidase, elevation of the expression of SIRT1 proteins, increases in the cellular mitochondrial membrane potential and NO levels, inhibition of the p38MAPK/NF-κB pathway, and inhibition of the production of ROS (Figure 2B).

### 3.4 Regulation of vascular function

Vascular dysfunction is a common feature of CVD (68). Sea buckthorn flavonoids protect against ox-LDL-induced endothelial cell damage by regulating the expression of eNOS and LOX-1. This result was also confirmed for quercetin and isorhamnetin (40). The polyphenolic compounds extracted from sea buckthorn berries were shown to increase the activity of antioxidant enzymes and mitigate the expression of ICAM-1, eNOS and LOX-1 in the aortas of hyperlipidemic rats. This resulted in a reduction in vascular endothelial damage (49). Another study revealed that sea buckthorn fruit also reduced the expression of iNOS mRNA and protein in the rat aorta and protected against hyperlipidemia-induced vascular endothelial dysfunction by regulating eNOS/NO (50). Zhu et al. treated rat vascular smooth muscle cells with sea buckthorn flavonoids, specifically quercetin and isorhamnetin. The findings indicated that the administration of the drug resulted in the blockade of VDC and ROC, which subsequently influenced the levels of [Ca^2+^](i) in vascular smooth muscle (41). In addition, feeding sea buckthorn powder cubes to spontaneously hypertensive rats reduced the MAP, heart rate, the plasma TC, TG and glycosylated hemoglobin levels. A reduction in hypertension affects enzyme expression in endothelial cells, and certain endothelial cells are converted from alkaline phosphatase (AP) to dipeptidylpeptidase IV (DPPIV) after sea buckthorn treatment (25).

In summary, sea buckthorn ameliorates vascular endothelial and smooth muscle cell injury. Among the sea buckthorn flavonoids, both isorhamnetin and quercetin exhibited this effect. Specifically, they may regulate vascular function by modulating the expression of eNOS, iNOS, and LOX-1 mRNA and protein, decreasing ICAM-1 expression, decreasing the level of [Ca^2+^](i), and affecting the expression of enzymes in endothelial cells (Figure 2C).

### 3.5 Anti-platelet aggregation effects

Platelet activation is a key factor in AS and thrombosis. During the development of AS, platelets activate and aggregate in the arterial wall, promoting thrombus formation and extension (69). Leaf extract and sea buckthorn seed oil inhibited platelet hyperactivity (70). The results of one study revealed that compounds isolated from the phenolic fraction of sea buckthorn berries reduce lipid peroxidation and protein carbonylation in human plasma and interfere with the thrombin receptor in platelets to regulate platelet activation (57). Both compounds also inhibit collagen-stimulated platelet activation and P-selectin exposure in stimulated platelets (58). Phenolic extracts from sea buckthorn leaves and twigs exhibit anti-adhesive properties by diminishing platelet binding to collagen and fibrinogen. Moreover, the phenolic extract derived from the twigs effectively inhibits the enzymatic pathway of arachidonic acid metabolism in platelets triggered by thrombin. Compared with leaf extracts, twig phenolic extracts promoted better inhibition of platelet adhesion to type I collagen (34). Phenolic compounds found in sea buckthorn fruits have been shown to inhibit the oxidative damage caused by H_2_O_2_ or H_2_O_2_/Fe to human plasma lipids and the carbonylation of human plasma proteins. It also regulates ROS production by interfering with the metabolism of arachidonic acid (46). In another experimental study, the researcher showed that a phenolic extract reduced the level of ROS in thrombin-activated platelets, inhibited the hydrolytic properties of thrombin proteins, and inhibited the activation cascade downstream of G proteins in platelets (47).

**Figure 3:**
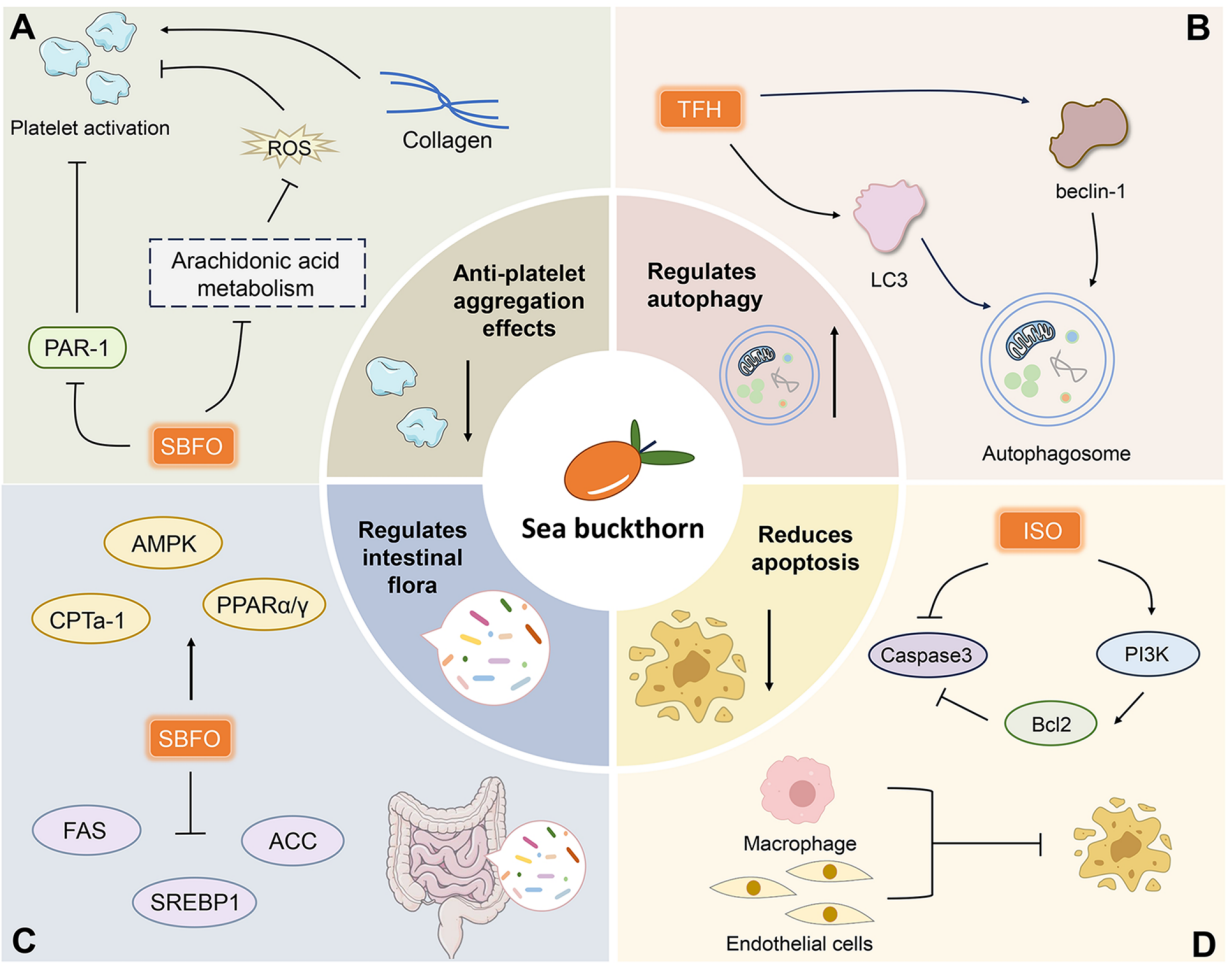
Mechanisms of action of anti-platelet aggregation **(A)**, regulation of autophagy **(B)**, regulation of intestinal flora **(C)** and reduced apoptosis **(D)** of sea buckthorn active ingredients. PAR-1, protease-activated receptor-1; FAS, fatty acid synthase; SREBP-1, sterol-regulatory element binding protein 1; ACC, acetyl-CoA carboxylase; LC3, light chain 3; Bcl2, B-cell lymphoma-2.

In summary, sea buckthorn can inhibit platelet activation or aggregation by interfering with thrombin receptors on platelets, inhibiting collagen, inhibiting arachidonic acid metabolism, and decreasing ROS levels (Figure 3A).

### 3.6 Regulation of autophagy

Autophagy is a mechanism of protein digestion and organelle activity that occurs in cytosolic lysosomes. Autophagy occurs when intracellularly damaged components are removed from the cell, and furthermore, endothelial cells are resistant to stimulation by inflammatory molecules (71). Sea buckthorn flavonoids increase the protein levels of beclin-1 and LC3 in rats with AS and induce autophagy to inhibit the development of AS (42). In addition, the active substances extracted from sea buckthorn berries had a protective effect against damage caused by I/R in isolated rat hearts. Treatment with sea buckthorn fruit extract improved myocardial function in rats and reduced the extent of cardiomyocyte damage by decreasing the leakage of LDH and CK-MB in the coronary effluent. Moreover, sea buckthorn berry active substances inhibited the protein levels of beclin-1 and LC3, suggesting that the protective effect of sea buckthorn active substances on isolated MIRI in rats may be related to autophagy (48).

Most findings suggest that sea buckthorn inhibits the development of CVD by regulating the autophagy proteins beclin and LC3. The detailed mechanism by which sea buckthorn occurs in organisms to treat CVD during autophagy needs to be further investigated (Figure 3B).

### 3.7 Regulation of the intestinal flora

Gut microbes are involved in the development of various diseases. There is a robust correlation between gut microbial imbalances and the onset and progression of CVD (72). Experimental studies have shown that sea buckthorn polysaccharides effectively increase the abundance of the intestinal flora in zebrafish, increasing the number of healthy and beneficial genera for the intestinal system, enhancing the recovery of dysbiotic intestinal flora and maintaining the balance of the system (52).

Sea buckthorn fruit oil increased the response to free fatty acid oxidation and catabolism (AMPK, CPTa-1, PPARα, and PPARγ) while inhibiting the synthesis of TG and fatty acids (SREBP-1, FAS, and ACC) at the gene and protein levels. In addition, sea buckthorn fruit oil intervention enriched the intestinal flora of hyperlipidemic mice by increasing the relative abundance of *Lactobacillus*, *Faecalibaculum*, and *Allobaculum* while decreasing the proportion of *Firmicutes/Bacteroidetes* (30). Sea buckthorn lyophilized powder regulates the intestinal microbiota, increases the abundance of some intestinal bacteria, upregulates genes involved in lipid synthesis and accumulation, and upregulates genes involved in lipolysis to inhibit fat accumulation (26).

These findings imply that sea buckthorn may impact CVD by changing gut microbes and offer novel approaches to treating CVD in sea buckthorn (Figure 3C).

### 3.8 Reducing apoptosis

Apoptosis maintains the stability of organisms, and an imbalance in apoptosis often leads to the development of diseases, including CVD (73). The administration of isorhamnetin inhibited the accumulation of apoptotic macrophages in mice, as evidenced by a reduction in caspase-3 expression and a decrease in the number of TUNEL-positive cells (56). These findings suggest that isorhamnetin attenuates AS through apoptosis. von Willebrand Factor (vWF) and Thrombomodulin (TM) are molecular markers of endothelial cell injury, and abnormal levels of vWF and TM occur when the endothelium is dysfunctional. Sea buckthorn flavonoids significantly attenuated vascular endothelial cell injury caused by adrenaline combined with ice bath establishment, and reduced the plasma levels of vWF and TM (74). In addition, phenylpropyl compounds extracted from sea buckthorn were able to exhibit protective effects against myocardial injury in zebrafish by decreasing the expression of caspase-3 (43).

In summary, sea buckthorn can attenuate CVD by decreasing Caspase-3 expression and the number of TUNEL-positive cells, and activating the PI3K/Bcl-2/Caspaes-3 pathway (Figure 3D).

## 4 Clinical applications

The mechanism of action of sea buckthorn as a potential drug for the treatment of CVD has been revealed in preclinical studies. To further investigate the efficacy and specific mechanism of action of sea buckthorn in the clinic, sea buckthorn has been made into different preparations for clinical trial studies. In this chapter, we review the clinical studies of sea buckthorn in the treatment of CVD and find that sea buckthorn is effective in improving lipid metabolism, hypertension, and antithrombosis in patients (Table 2).

**Table 2:** Clinical applications of sea buckthorn in CVD.

Study design	Subject	Trial drug	Drug dose	Intervention period	Main results	Reference
Double-blind, controlled, randomized	229 healthy females and males	Sea buckthorn berry	28 g/day	90 days	Decrease in circulating concentrations of C-reactive protein	(75)
Crossover, randomized	80 overweight or mildly obese women	Dried sea buckthorn berries, sea buckthorn oil, sea buckthorn phenolics ethanol extract mixed with maltodextrin (1:1), frozen bilberries	20 g/day, 4 g/day, 14.6 g/day	30 days	Decreases TG and VLDL subclasses; decreases TC, LDL-C and apolipoprotein B; increases TG and VLDL	(76)
Single-blind, controlled, randomized	116 patients with hypertension	Sea buckthorn flavonoid	10–30 mg three times a day	4 months	Decreased blood pressure; decreased 24-h urinary protein, blood β2-MG and urinary β2-MG	(77)
Double-blind, controlled, randomized	32 healthy and 74 patients with hypertension and hypercholesterolemia	Sea buckthorn seed oil	0.75 ml/day	30 days	Decreased blood pressure; decreased cholesterol, oxy-LDL and TG	(78)
Double-blind, controlled, randomized	11 healthy males	Sea buckthorn berry oil	5 g/day	4 weeks	Reduced aggregation reaction and maximum aggregation rate	(79)
Double-blind, controlled	20 male non-smokers aged 18–55 years	Sea buckthorn juice	300 ml/day	8 weeks	20% and 17% increase in HDL-C and TG concentrations	(80)
Double-blind, crossover, randomized, dose-escalation	7 females and 6 males	Sea buckthorn oil supplemented with cis-palmitoleic acid (16:1n-7c) or trans-palmitoleic acid (16:1n-7t)	Supplementation with 16:1n-7c (380, 760 and 1,520 mg/day) and 16:1n-7t (120, 240 and 480 mg/day) 3 weeks	22 weeks	Phospholipids increased by 26.6% at the highest dose supplemented with 16:1n-7t	(81)
Two-stage, randomized crossover and double-blind	38 patients with impaired glucose regulation	Sea buckthorn fruit puree	90 ml/day	5 weeks	Fasting blood glucose decreased	(82)

Sea buckthorn has been made into different preparations for use in CVD. Xindakang tablets are a CVD therapy medicine whose major constituent is sea buckthorn flavonoids (83). It is a natural medicine for the treatment of CVD developed over the years by a team from China's West China University of Medical Sciences and is listed as a protected national variety of traditional Chinese medicine. Xindakang tablet has been clinically demonstrated to be effective over a longer period of time. Xindakang tablets play a role in the treatment of coronary angina through multi-components, multi-targets, multi-pathways, and can inhibit inflammation, oxidative damage, apoptosis, and reduce coronary artery spasm and coronary artery stenosis (84). A 3-month study of 200 outpatients on a patent similar to the active ingredient in Xindakang showed that the preparation exhibited a therapeutic effect on angina pectoris (83).

Abnormal lipid metabolism represents a significant risk factor for the development of CVD. A clinical trial, employing a randomized, double-blind design, was carried out with 229 individuals in good health, ranging in age from 19 to 50 years. During the 90-day trial, the subjects were allocated randomly to ingest 28 g of sea buckthorn juice or a placebo daily. The results revealed an increase in the levels of isorhamnetin and quercetin in the blood, along with a significant decrease in the amount of C-reactive protein in the blood of participants after they consumed sea buckthorn berries. However, no correlation was observed between these changes. Furthermore, sea buckthorn berries do not affect the concentration of lipids in the participants’ blood (75). This is somewhat different from the results of other trials. In a further randomized crossover trial, 80 overweight women consumed dried sea buckthorn berries, oil, phenolic extracts mixed with maltodextrin (1:1), or frozen lingonberries for a period of 1 month. Dried sea buckthorn fruits affect the serum TG and VLDL subclasses, sea buckthorn oil decreases the total serum cholesterol, IDL and LDL-C levels; and sea buckthorn phenolic extracts mixed with maltodextrin increase the serum TG and VLDL levels (76). The ingestion of sea buckthorn has been demonstrated to enhance lipid metabolism in individuals with underlying health issues, yet it has no discernible effect on lipid status in those who are generally healthy. Therefore, people can consume sea buckthorn as a normal nutritional supplement (85).

Pilot studies suggest that sea buckthorn improves vascular endothelial function and reduces calcium ion levels in vascular smooth muscle. In a systematic evaluation of sea buckthorn flavonoids in essential hypertension, seven clinical trials were included and systematically evaluated patients in terms of systolic blood pressure, diastolic blood pressure, posterior wall thickness of the left ventricle, interventricular septal thickness, improvement in renal function, and adverse drug reactions. Studies have shown that sea buckthorn flavonoids alone are comparable to calcium channel blockers (CCBs) and ACEIs in reducing diastolic blood pressure and are weaker than ACEIs in reducing systolic blood pressure, and there is no significant difference between CCBs and ACEIs. The combination of sea buckthorn flavonoids and conventional treatment was more effective than conventional treatment in reducing left ventricular hypertrophy. Hypertension is closely related to the deterioration of renal function. Sea buckthorn flavonoids are comparable to ACEIs in lowering 24-h urinary protein and blood β2-microglobulin, and improving creatinine clearance. In addition, sea buckthorn flavonoids have fewer adverse effects than ACEIs do (77). A randomized controlled double-blind study was conducted on 32 healthy subjects and 74 subjects with hypertension and hypercholesterolemia. The samples were supplemented with 0.75 ml of sea buckthorn seed oil daily for 30 days. The results demonstrated that this intervention normalized systolic and diastolic blood pressure and significantly reduced cholesterol, oxy-LDL, and TG in subjects with hypercholesterolemia. This correlation could be attributed to the high contents of linolenic acid, linoleic acid, and oleic acid found in sea buckthorn seed oil. Interestingly, in healthy individuals, sea buckthorn seed oil had no discernible impact on cholesterol or blood pressure (78).

Sea buckthorn plays an active role in anti-platelet aggregation. An experiment was carried out using a small double-blind randomized controlled trial involving 11 healthy adult males. They were administered 5 g of sea buckthorn berry oil daily for a period of 4 weeks, with coconut oil serving as the control substance. The results indicated that sea buckthorn berry oil did not affect plasma or platelet phospholipid fatty acids but decreased the rate of ADP-induced aggregation reactions and the maximum aggregation rate. Furthermore, the subject's weight was not significantly affected by sea buckthorn berry oil. These results indicate that sea buckthorn berry oil is valuable in the treatment of blood coagulation, and the next step could be to investigate the specific mechanisms to clarify the role of sea buckthorn berry oil in actual treatment (79). Furthermore, a double-blind controlled trial was conducted, in which 20 adult males without underlying health issues were recruited to consume 300 ml of sea buckthorn juice daily for a period of 8 weeks. The levels of plasma lipids, ox-LDL, platelet aggregation and plasma soluble cell adhesion protein were quantified. The results revealed no statistically significant alterations in plasma TC, LDL-C, platelet aggregation, or ICAM-1 levels in the treatment group. However, there was a 20% increase in the HDL-C concentration and a 17% increase in the TG concentration (80). These experimental results point to the potential role of sea buckthorn in reducing CVD incidence, but the corresponding clinical evidence is scarce, and there are discrepancies in study design and trial results. There is still a need for further exploration to solve these problems and explain the in-depth mechanism of sea buckthorn action.

Sea buckthorn has a regulatory effect on blood glucose levels in the body. After sea buckthorn is consumed, the metabolic concentration of flavonols in the blood plasma remains high for several hours, which provides the body with a stable supply of flavonols (86). To determine the dose-escalation effects of supplementing with sea buckthorn oil containing cis- and trans-palmitoleic acid (16:1n-7c and 16:1n-7t) on the serum phospholipid fatty acids (PLFA) content of 17 participants, a randomized, double-blind, crossover, dose-escalation trial was carried out. The supplementation of sea buckthorn oil with 16:1n-7t resulted in a dose-dependent increase in PLFA, whereas the supplementation of unmodified sea buckthorn oil also yielded a corresponding increase in PLFA. These findings suggest a potential role for this isomer in metabolic diseases, including those affecting glucose metabolism (81). Zhong et al. conducted a two-phase randomized, crossover, controlled and double-blind intervention trial to investigate the effects of a sea buckthorn whole fruit mixture on fasting and postprandial glycemic responses in patients with impaired glucose regulation (IGR). At the end of the trial, fasting blood glucose in IGR patients was reduced by 0.13 ± 0.56 mmol/L and was able to produce positive effects up to 28 days after the end of the trial (82). This evidence suggests that sea buckthorn has a modulating effect on blood glucose levels, but the relevant literature and the number of participants are limited, and there is still a need for a sufficient number of trials to demonstrate its efficacy.

## 5 Safety and toxicity

Sea buckthorn acts as medicinal plants, and assessing their safety is particularly important. The rats were given 100, 250, or 500 mg/kg of sea buckthorn extract daily; after 90 days of therapy, rats supplemented with 250 or 500 mg/kg of sea buckthorn extract showed a substantial increase in plasma glucose levels, which returned to normal 2 weeks after treatment ended. No adverse reactions were observed in the sea buckthorn extract 100 mg/kg dose group. Therefore, it was used as a NOAEL for sea buckthorn extract in rats (87). In the acute toxicity investigation, the highest amount of sea buckthorn oil that the mice could tolerate was >20 ml/kg, whereas the NOAEL for the rats was 10 mg/kg. This oral toxicity study had a 90-day trial period, during which no fatalities or toxicological symptoms were observed (88). Another study examined the genotoxic and teratogenic effects of sea buckthorn oil. Genotoxicity studies revealed no mutagenic activity of sea buckthorn oil on histidine-dependent strains of *Salmonella typhimurium*, no effect on sperm morphology, and no effect on the micronucleus rate of polychromatic erythrocytes in mice. Teratogenicity studies have revealed that sea buckthorn oil is not toxic to pregnant rats or their embryos (89). Studies on the acute and subacute oral toxicity of supercritical CO2-extracted sea buckthorn seed oil in treating burn wounds in rats have demonstrated the absence of any significant toxicity or adverse effects associated with the use of this oil (90). Saggu et al. reported no serious toxicity of sea buckthorn leaf extract on any of the vital organs of rats with a maximum effective adaptogen dose of 100 mg/kg body weight. The LD_50_ is the dose of a drug that kills half of the animals. This study revealed that the LD_50_ value of sea buckthorn leaf extract was >10 g/kg body weight (91). *In vivo* toxicity assessment of the oral administration of herbal antioxidant supplement (HAOS) from sea buckthorn revealed that HAOS did not cause any symptoms of death or toxicity at selected oral doses. In addition, HAOS increased the bioavailability of vitamin A and vitamin C in humans by 32% and 172%, respectively (92). Sea buckthorn-based herbal oil (a blend of sea buckthorn seed oil, pulp oil, and pomace oil at a ratio of 30:35:35) was used in a 14-day UV irradiation stimulation test on rabbit skin by Nishad et al. They reported that at 72 h the herbal oil completely eliminated the faint erythema and edema produced by UV exposure. These findings suggest that this herbal oil is non-irritable to rabbit skin and is safe and effective (93). Sea buckthorn has a protective effect against sulfur dioxide and mustard gas-induced injury. The organs (liver, lungs, kidneys, spleen, etc.) to body weight ratio changed and the number of micronuclei in polychromatic erythrocytes increased in mice after inhalation of sulphur dioxide. Acceptance of intraperitoneal injection of sea buckthorn seed oil reduced to some extent the damage to organs and genetic material in mice (94). Ethanolic and aqueous extracts of sea buckthorn showed protective effects against transdermal administration of mustard gas to mice, whereas ethanolic extracts of sea buckthorn leaves and sea buckthorn flavonoids had significant effects with indices of 2.4 and 2.2, respectively. Notably, the levels of GSH, GSSG, and MDA were restored after the oral administration of ethanolic extracts of sea buckthorn leaves and sea buckthorn flavonoids (95). Arsenic, a non-metallic element widely distributed in the environment, including air, water and land, is highly toxic. The prevention and control of arsenic poisoning are urgently needed. For 3 months, the mice were administered aqueous and ethanol extracts of sea buckthorn and were exposed to drinking water contaminated with arsenic. The findings indicated that a 500 mg/kg body weight concentration of sea buckthorn aqueous extract considerably reduced the oxidative damage caused by arsenic. However, it does not chelate arsenic (96).

## 6 Conclusion

Sea buckthorn has been widely studied because of its nutritional value and medicinal value, which is limited not only to the fruit but also to its flowers, leaves, stems, roots and other parts, which have high medicinal value and economic value. Sea buckthorn contains flavonoids, tannins, terpenoids, polysaccharides, vitamins, and other active ingredients, which endow sea buckthorn with a variety of pharmacological effects, including anti-inflammatory (97), antioxidant (98), hepatoprotective (99), anticardiovascular (100), antiaging (101), immunity-regulating (102), antitumor (103), and antibacterial (104) effects. Flavonoids are the most widely studied active ingredients in sea buckthorn, and more than 90 flavonoids have been extracted and isolated from sea buckthorn. The most important compound, isorhamnetin, potentially plays a role in the treatment of CVD. Isorhamnetin, a hot research topic in recent years, can exert anti-inflammatory and anti-platelet aggregation effects through different signaling pathways such as the PI3K/AKT pathway (102). The use of monomeric compounds such as kaempferol and quercetin is another direction for the study of the active components of flavonoids. However, the study of sea buckthorn flavonoids is still limited to monomer compounds, the derivatives of the monomer compounds have not been studied in depth, and there is a lack of data supporting their chemical modifications and conformational relationships. Therefore, in subsequent studies, sea buckthorn compounds can be further developed and utilized on the basis of their pharmacodynamic properties.

Preclinical studies have shown that sea buckthorn has anti-inflammatory, lipid oxidation modulating, antiplatelet aggregation, antioxidant, vascular function modulating, autophagy, gut microbial modulating, and apoptosis reducing effects. The pharmacological effects of sea buckthorn involve multiple sites and multiple components. However, these pharmacological effects lack synergistic effects and interactions with traditional cardiovascular drugs. Therefore, additional investigations are needed to examine the function of sea buckthorn in all-encompassing treatment plans. In addition, sea buckthorn was found to improve lipid metabolism in patients with dyslipidemia and to have no effect on blood lipids in healthy individuals in clinical trials. Sea buckthorn was able to normalize blood pressure in hypertensive patients, again with no significant effect on blood pressure in healthy subjects. However, many studies on sea buckthorn are still in the laboratory stage and preliminary clinical trials, and there is a lack of large-scale and multicenter clinical trials, which cannot fully reflect the specific efficacy of sea buckthorn and increase the difficulty of subsequent in-depth research, limiting the application and promotion of sea buckthorn in practical medical treatment. Therefore, more clinical trials are urgently needed in the future as strong evidence to provide effective information for the further application of sea buckthorn.

The results of the present study revealed that no significant toxicity was found with the doses of sea buckthorn used in the experiment. It also has protective effects on different organs of experimental animals in hazardous environments, such as ultraviolet radiation, sulfur dioxide stimulation and arsenic exposure. These findings suggest that sea buckthorn may not have toxic effects on the human body at the recommended dose, which is safe and reasonable. However, this does not ensure that sea buckthorn has no adverse effects on the human body, and people still need to take sea buckthorn at a safe dose to ensure its safety and efficacy.

Sea buckthorn offers enormous potential in the treatment of CVD. In the future, additional efforts should be made to explain the interactions between the active components in sea buckthorn and CVD targets, in order to better understand the mechanism of action of sea buckthorn in CVD treatment. The active ingredients in sea buckthorn do not exert their therapeutic effects individually, and the synergistic effect of the active ingredients can be further investigated. In addition to its present applications in hyperlipidemia and hypertension, the efficacy of sea buckthorn in other disorders should be investigated further in order to broaden the area of therapeutic applications and provide more options for therapy. Meanwhile, particular research on sea buckthorn treatment should be conducted for patients of all ages, genders, and races, as well as patients with other underlying conditions, in order to clarify the specific efficacy and safety of sea buckthorn in all populations. New dosage forms of sea buckthorn can be developed in drug research and development, such as slow-release preparation, controlled-release preparation, and nano-preparation, to improve the drug's stability and bioavailability, reduce the number of times it is administered, and improve patient adherence. By developing targeted medication delivery technology, sea buckthorn can work more precisely on the lesion area, reducing the adverse reaction of other organs and improving the therapeutic efficacy. In addition to sea buckthorn, salvia and *Phyllanthus embilica L.* have been used as traditional Chinese herbs in the prevention and treatment of CVD. Similarly, Salvia also plays a protective role against CVD in terms of intestinal flora and autophagy (105, 106). Of course, the active ingredients of the three differ and the target mechanism of action is also different (107, 108). This also provides ideas and references for the subsequent prevention and treatment of sea buckthorn in CVD.

To summarize, this paper reviews the mechanism of action, clinical research and safety of sea buckthorn in CVD, with the hope that sea buckthorn can be more safely and effectively applied in the clinic for the treatment of CVD.
